# Global snapshot of the effects of the COVID-19 pandemic on the research activities of materials scientists between Spring and Autumn 2020

**DOI:** 10.1080/14686996.2021.1894756

**Published:** 2021-04-21

**Authors:** Adarsh Sandhu, Roland Hany, Atsufumi Hirohata, Shunichi Hishita, Ken Kimlicka, Masanobu Naito, Chikashi Nishimura

**Affiliations:** a Department of Engineering Science, Graduate School of Information and Engineering, University of Electro-Communications, Tokyo, Japan; b Lab for Functional Polymers, Empa, Dübendorf, Switzerland; c Department of Electronic Engineering, University of York, York, UK; d Materials Data Platform Center, Publishing Team, Research and Services Division of Materials Data and Integrated System, NIMS, Tsukuba, Japan; e Global Head of Portfolio, Taylor & Francis, Tokyo, Japan; f Data-driven Polymer Design Group, Research and Services Division of Materials Data and Integrated System, NIMS, Tsukuba, Japan

**Keywords:** COVID-19, materials science, scientific research, pandemic, lockdown, virtual conference, COVID-19 survey, science careers, crisis management, research management, General, 70 New topics/Others

## Abstract

We conducted a global survey on the effects of the COVID-19 pandemic on the research activities of materials scientists by distributing a questionnaire on 9 October 2020 with a response deadline of 23 October 2020. The questions covered issues such as access to labs, effectiveness of online conferences, and effects on doctoral students for the period covering the first lockdowns until the relaxation of restrictions in late September 2020 in many countries. The survey also included online interviews with eminent materials scientists who shared their local experiences during this period. The interviews were compiled as a series of audio conversations for *The STAM Podcast* that is freely available worldwide. Our findings included that the majority of institutes were not prepared for such a crisis; researchers in China, Japan, and Singapore were able to resume research much quicker – for example after approximately one month in Japan – than their counterparts in the US and Europe after the first lockdowns; researchers adapted to using virtual teleconferencing to maintain contact with colleagues; and doctoral students were the hardest hit by the pandemic with deep concerns about completing their research and career prospects. We hope that the analysis from this survey will enable the global materials science community to learn from each other’s experiences and move forward from the unprecedented circumstances created by the pandemic.

## Graphical Abstract

**Figure:**
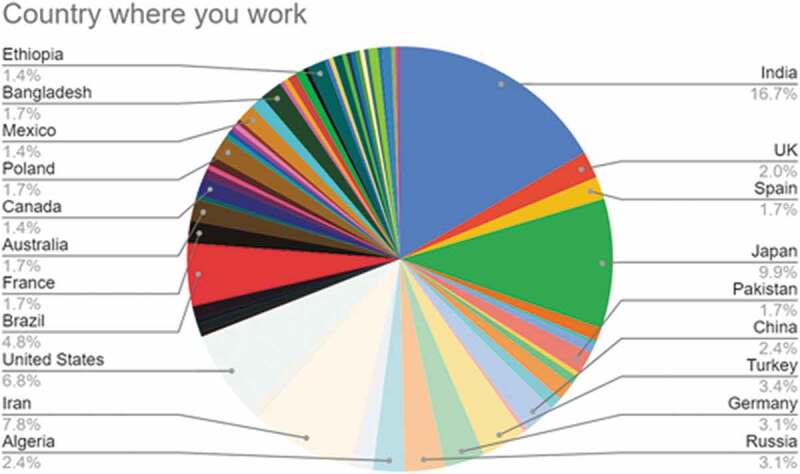

## 1. Introduction

As of writing this manuscript in mid-January 2021 the spread of the COVID-19 coronavirus (SARS-CoV-2) pandemic has led to approximately 94 million reported cases and 2 million deaths globally [1]. Since the first reported cases in China in late 2019 to the approval and role of vaccines in December 2020 the COVID-19 outbreak has caused unprecedented disruption to our lives on every continent including most recently, Antarctica with reports in late December 2020 of 36 cases at Chile’s Bernardo O’Higgins research station on the Antarctic Peninsula [2]. The term ‘lockdown’ is no longer just the realm of dystopic movies depicting destruction unleashed by imagined diseases and conflicts. In the real world, the spread of COVID-19 has been covered by 24-h news channels offering real-time, high resolution, multilingual, front-seat views of the chaos inflicted by COVID-19 on the lives of myriads of people across the world [3]. Perhaps understandably, the plight of people focussed on scientific research has been a low priority for newspaper journalists and headline writers. But just like many other members of society, COVID-19 has dramatically affected the lives of scientists and how they conduct science. Articles on ‘research and COVID-19’ have been published in scientific journals with many reports focussing on the impact of COVID-19 on researchers in biomedical sciences and healthcare [4–7]. Surprisingly, and to the best of our knowledge, there have not been any significant in-depth reports on the effects of the COVID-19 pandemic on research being undertaken by materials scientists and engineers.

Here, we report the results of an extensive global survey on how the spread of the COVID-19 coronavirus and resulting restrictions on mobility affected the lives and research activities of materials scientists following the first wave of lockdowns in the Spring of 2020. We implemented two types of surveys: (1) A questionnaire sent by direct mail to materials scientists worldwide on 9 October 2020 with a deadline of 23 October 2020. The questions covered issues affecting materials scientists during the initial lockdowns in the Spring of 2020 until relaxation of restrictions in late September 2020; (2) Direct videoconference interviews with eminent materials scientists including Professor Katsuhiko Ariga of the National Institute for Materials Science (NIMS) in Japan [https://podcasts.apple.com/us/podcast/katsuhiko-ariga-materials-research-education-covid/id1517265384?i=1000483425553], Professor Hong Lin of Tsinghua University, Beijing [https://podcasts.apple.com/podcast/the-stam-podcast/id1517265384], Professor James K. Gimzewski of University of California, Los Angeles [https://podcasts.apple.com/us/podcast/james-k-gimzewski-materials-research-education-covid/id1517265384?i=1000491233350], and Professor Daniel Ponce of Universidad de Cádiz/IMDEA Nanoscience, Spain. The interviews were compiled as a series of ‘*STAM Podcasts*’ that are freely accessible on Apple Podcasts [8], Google Podcast [9] and Spotify [10].

Our survey showed that the materials science community is robust and adapted well during the early days of the pandemic despite sudden lockdowns and inaccessibility to research laboratories and equipment. The hardest hit were doctoral students who expressed deep concerns about completing their research and career prospects.

## 2. Methodology and implementation of the surveys

### 2.1. Questionnaire (complete list in supplementary information 1)

The questionnaire entitled ‘Survey on the effects of the COVID-19 pandemic on research activities of materials scientists’ covered general issues such as location, gender, age group and areas of research. It also delved into matters more pertinent with materials science such as maintenance of equipment during lockdown; methods for communicating with colleagues and effectiveness of virtual conferences; availability of extra-funding for restarting research; physical and mental health; and effects of the crisis on doctoral students. The survey was carried out by distributing emails with an embedded link to our Google Forms questionnaire to the following recipients: (1) our Science and Technology of Advanced Materials (STAM) Editorial Office mailing list of 2,000 people who attended events organized by STAM in Japan, USA, and Europe. The response rate from this group was 0.5%; (2) Members of an international audience of academics and graduate school students at a university in Japan, where we received 75 responses; (3) A direct mail campaign implemented using a database provided by Taylor and Francis (T&F), where 32,333 emails were successfully delivered to materials scientists worldwide, yielding 652 unique clicks (click through rate of 2%), which is typical for such campaigns. The T&F campaign yielded a total of 298 responses from a global distribution of respondents located in 35 countries. The responses were collected automatically with the Google Forms System and analysed as described below.

### 2.2. Interviews and the STAM Podcast

The Google Forms email survey was complemented with interviews of eminent materials scientists to gain direct insights into issues affecting them at the time of our conversations. The interviews were conducted using video teleconferencing technology and the results were compiled as a series of *STAM Podcasts* described as ‘Materials research, education, and COVID-19: In conversation with materials scientists about the unique challenges facing materials scientists as they assess the impact of the unprecedented changes triggered by COVID-19’. Details about the interviewees can be found by visiting the *STAM Insights* website (https://stam-insights.e-materials.net/).

Notably, the first interview was conducted on 27 May 2020 with a scientist in the UK and the tenth and most recent, was recorded on 9 December 2020 with a researcher at an Australian university. The conversations reflect and are synchronised with the extensively reported ‘waves’ of the spread of the pandemic, beginning with the hard lockdowns declared in the Spring of 2020, followed by relaxations of restrictions on movement during Summer and Autumn, and the recent reintroduction of tough lockdown rules towards the end of 2020. The timeline and list of interviewees are available in Supplementary information 2.

## 3. Results and analysis

### 3.1. Responses to general questions

Figures 1 to 6 shows the responses to the general questions of the survey.

#### 3.1.1. Country where you work

**Figure 1.**
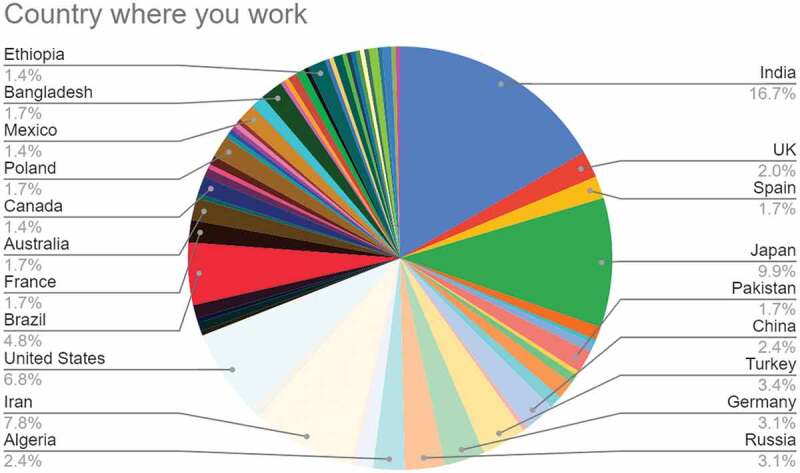
Chart showing selected responses to the question ‘Country where you work’

#### 3.1.2. Gender

**Figure 2.**
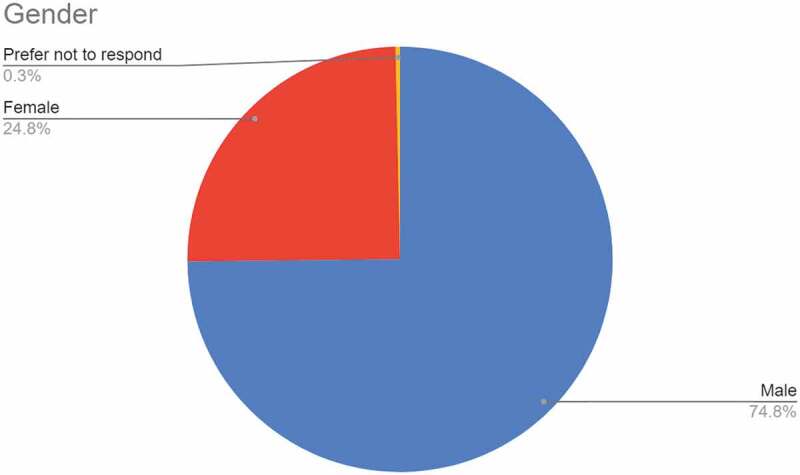
Response to the question on gender

#### 3.1.3. Age group

**Figure 3.**
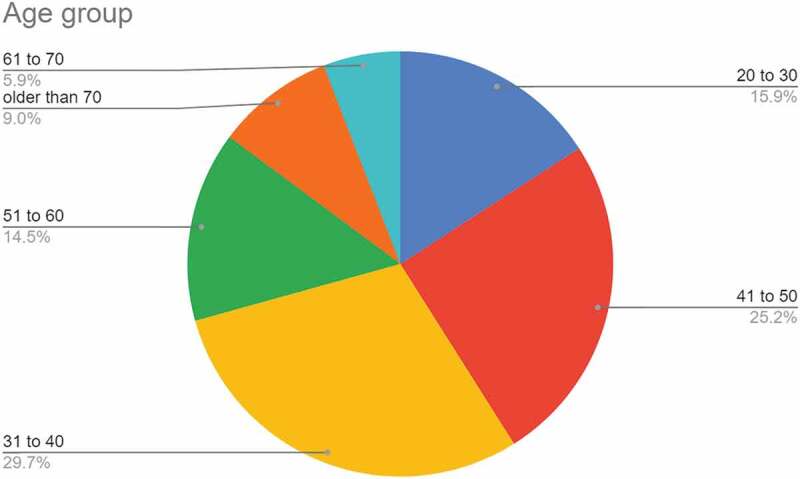
Chart showing responses to the question on age group

#### 3.1.4 Your institute

**Figure 4.**
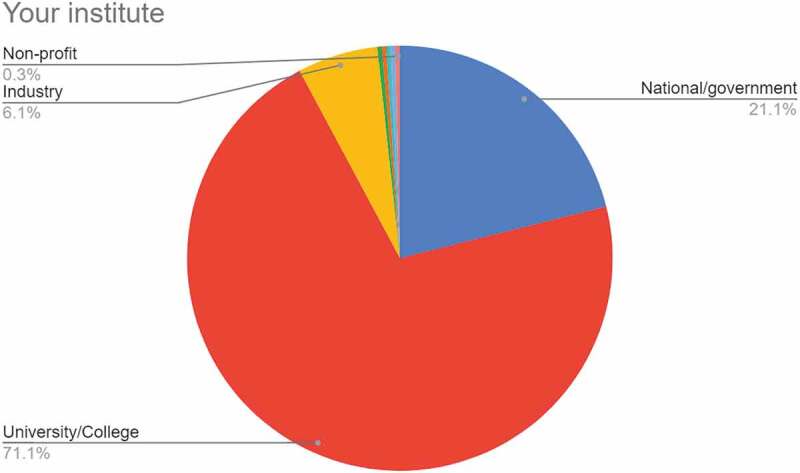
Chart showing responses to the question ‘Your institute’

#### 3.1.5. Your current position

**Figure 5.**
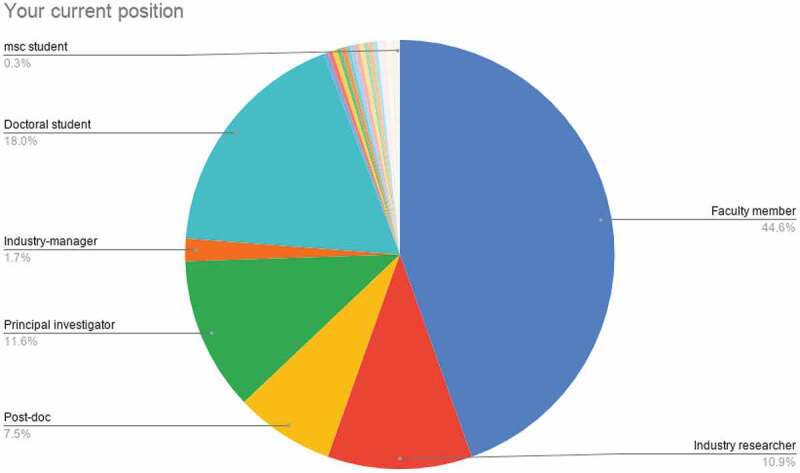
Chart showing responses to the question ‘Your current position’

Note for clarifying the legends of the chart.

1. ‘Faculty member’ includes professor, associate professor, and assistant professor.
2. ‘Industry researcher’ includes ‘Industry scientist’.
3. ‘Principal investigator’ refers to respondents based at research institutes.

#### 3.1.6. Area(s) of research (check as many as appropriate)

**Figure 6.**
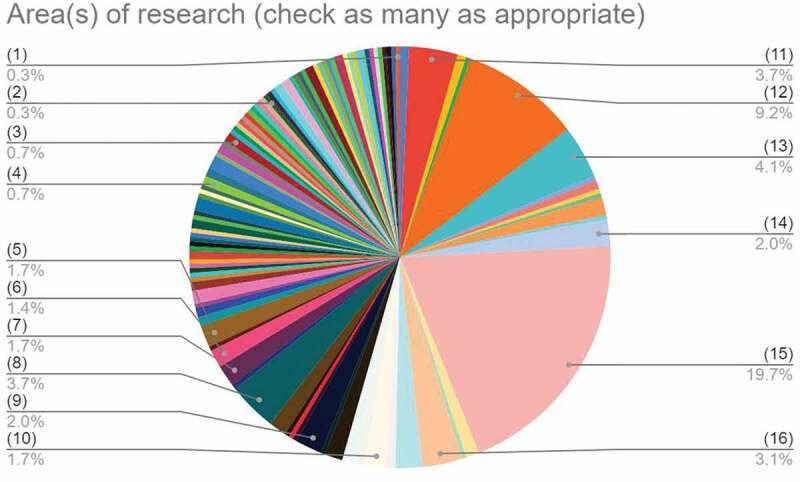
Chart showing selected responses to the question on ‘Area(s) of research (check as many as appropriate)’. Explanation of the numbers in brackets

1. Engineering and structural materials, Energy materials, Materials for energy and environment, next-generation photovoltaics, and green technologies
2. Organic and soft materials (colloids, liquid crystals, gels, polymers), Energy materials, Nanostructured/nanoscale materials and nanodevices, Materials for energy and environment, next-generation photovoltaics, and green technologies
3. Nanostructured/nanoscale materials and nanodevices, Materials for energy and environment, next-generation photovoltaics, and green technologies
4. Engineering and structural materials, Organic and soft materials (colloids, liquid crystals, gels, polymers), Nanostructured/nanoscale materials and nanodevices, Bio-inspired, biomedical, and biological materials; nanomedicine, and novel technologies for clinical and medical applications
5. Energy materials
6. Engineering and structural materials, Materials for 3D printing and additive manufacturing
7. Engineering and structural materials, Organic and soft materials (colloids, liquid crystals, gels, polymers)
8. Engineering and structural materials, Nanostructured/nanoscale materials and nanodevices
9. Advanced structural materials, materials for extreme conditions
10. Optical, magnetic and electronic device materials
11. Nanostructured/nanoscale materials and nanodevices
12. New topics/Other
13. Bio-inspired, biomedical, and biological materials; nanomedicine, and novel technologies for clinical and medical applications
14. Engineering and structural materials, Advanced structural materials, materials for extreme conditions
15. Engineering and structural materials
16. Organic and soft materials (colloids, liquid crystals, gels, polymers)

## 4. Details of notable findings in response to the questionnaire

Questions: *‘Was your institute prepared for the restrictions in movement?’* and *‘How did you manage maintenance of equipment and materials for your research?’*

The responses showed that 54% of institutes were not prepared or took considerable time to react and only 44% had contingency plans in place. Furthermore, 33% of respondents had to shut down all equipment and 76% were allowed to enter their labs to check equipment during lockdown. However, our survey showed significant regional differences for access to labs restricted during the first lockdown. Specifically, labs in the UK and parts of the USA, Spain, and India were not accessible for the whole of the first lockdown. In contrast, scientists in Japan and Singapore were able to access the labs within 1 ~ 2 months after the first lockdown. The STAM Podcasts contain detailed accounts by scientists located in 10 countries of the state of their research during the first lockdowns [8–10].

Question: *‘How did you maintain contact with your colleagues/research students during lockdown?’*

To stay connected with colleagues, an overwhelming 89% of respondents used video teleconferencing, and a surprisingly 10% were still able to maintain regular face-to-face interaction. Difficulties encountered in maintaining regular contact with colleagues included unstable internet (nearly 50%) and inability to have spontaneous ‘coffee time’ style meetings (ca. 44%) and 36% stated they suffered from fatigue after many hours of teleconferencing.

Questions: *‘Did you experience isolation due to a lack of interaction with your colleagues?’; ‘Has your institute offered support in overcoming any mental stress due to the lockdown?’;* and ‘*How did you manage your physical health?’*

As many as 40% of respondents felt isolation throughout the crisis, 25% initially, and nearly 32% did not feel isolation at all. Also, 57% of the respondents did not receive mental health support from their institutes and 40% said they did get such support.

Questions: *‘Have you attended any virtual academic conferences since the lockdown?’; ‘Do you agree that virtual conferences are an effective way to interact with colleagues?’;* and ‘*If necessary, please state your opinion about virtual conferences.’*

**Figure 7.**
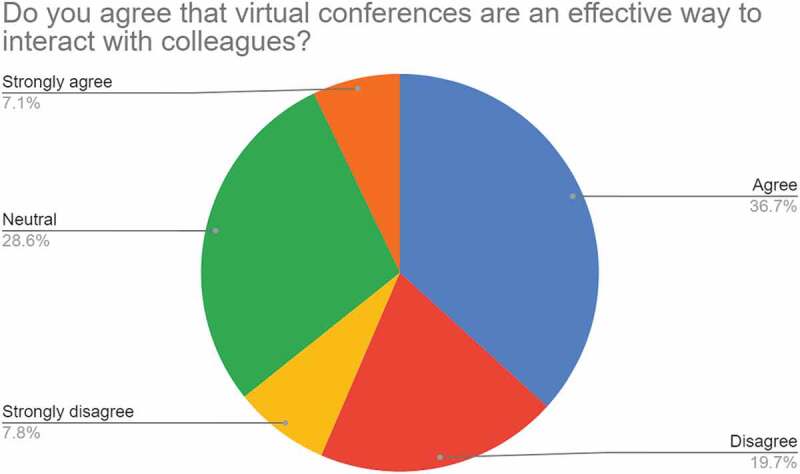
Response to ‘*Do you agree that virtual conferences are an effective way to interact with colleagues?’.*

Approximately 64% of respondents had attended virtual conferences, 35% had not, and as shown in Figure 7. only ca. 8% strongly disagreed with the statement that ‘*Do you agree that virtual conferences are an effective way to interact with colleagues?’*, indicating that virtual conferences were largely acceptable by the materials science community although as the comments below show, this was not without reservations.

A selection of other comments related to the effectiveness of virtual conferences during the lockdown:

- Not an effective way to have lively discussions with many people at different locations.
- A poor substitute. Small group talk on interests and opportunities are not possible … Personal interaction much more boosts creativity than screen meetings!
- The face-to-face communication is necessary.
- The conference is not just the place where you listen to others you get to interact and talk with them and exchange ideas and contacts.
- They are useless, because people need personal contact, because we are gregarious animals.
- Virtual conferences are tiring and boring.
- It is difficult to meet new people and change ideas, is completely different the personal contact.
- Good solutions to maintain exchanges.
- To keep academic communication they are necessary.
- Unfortunately, there is a lack of seriousness regarding virtual conferences as of now.
- Scientific interactions do not happen. It is a one-way communication with frequent disruptions and the purpose of conference is generally defeated.
- It can badly affect the interaction with people. I don’t think interaction during virtual conferences can be as effective as normally.
- There is no liveliness in the virtual conference.
- Unstable connection affects interaction in a virtual conference.
- They are cheaper, but lack creation of community of practice and connectedness.
- The major advantages of attending virtual conferences are saving money and time in all sorts like hotel reservation, flight tickets, transportation.
- While I still hear what others are researching, it is difficult to interact, ask questions, challenge each other, bounce ideas off each other, network, etc.
- We have different time between countries and the interaction is not the same as in the coffee break of a real conference.
- People are attentive. There are more questions raised at virtual conferences.
- While better than not having interactions, it is very hard to have in depth conversations with colleagues.
- It’s just a makeshift arrangement. The emotional connect is conspicuously lacking in these conferences. Coupled with network instability, its more stressful.
- Much easily organized with economical help. No need to make a trip.
- Virtual conferences should be supplemental but shouldn’t replace face-to-face conferences, keeping safe distancing and personal protection health protocols until situations are back to normal.
- It is quite difficult to establish new personal relationships at virtual conferences; they are effective for senior scientists but definitely ineffective for junior scientists and students.
- I prefer to listen than to talk, virtual conferences are nice, you can do other things when it gets boring!
- It is good, no need to go to conference physically. Real conference has also communication problem due to its limit of space and presentation time. I think there is almost no difference and virtual conference is very effective way of international meeting.
- Since the lockdown restrictions are not yet removed in some countries. And also, the stresses because of the pandemic is not yet solved. Because of this and many other unsolved issues, virtual conference is ideal way for commencing research conferences.
- It is very productive, especially for students studying in countries where there is no possibility for providing required funding of physical attendance. We could participate or even contribute in this manner.
- They are an effective alternative given the current situation, but not a long-term substitute – especially for an early-career researcher, wanting to make connections!
- Not worth attending.
- I believe that virtual conferences will have a perspective even in ordinary (non-extreme) conditions.
- In my point of view, virtual conferences do not effectively substitute face-to-face interactions with colleagues; however, it is the best option available for now and it is still very good that we have the opportunity to interact online and keep the work going on.

Question: *‘Please give a short description of any new scientific findings made during the lockdown period that are related to your research’*.

- *For experimentalists like us no work was done during the lockdown period, and hence primary progress was in the form of writing pending papers and analyzing existing data*; Stated research areas, “Nanostructured/nanoscale materials and nanodevices, Materials for energy and environment, next-generation photovoltaics, and green technologies, Advanced structural materials, materials for extreme conditions.
- Writing papers and book chapters by summarizing data collected before the lockdown.
- *Screening procedure based on simulations for exclusion criteria applied to clinical trials of magnetic hyperthermia for treating pancreatic cancer*; Stated research areas, Materials informatics and materials genomics, Nanostructured/nanoscale materials and nanodevices, Bio-inspired, biomedical, and biological materials; nanomedicine, and novel technologies for clinical and medical applications.
- New cocatalyst for hydrogen evolution.
- No significant finding was made during the lockdown period.
- I was able to carry out the bit lengthy computational experiments.
- I have time to study new research area during lockdown.
- *I have published 2 SCI papers during the lockdown*; Stated research areas, Engineering and structural materials, New topics/Other
- I sent my new article to the international science magazine and it was published in this time.
- *We were able to study 3-D printed metastructures and test their behavior under compression loading*; Stated research areas, Engineering and structural materials, Organic and soft materials (colloids, liquid crystals, gels, polymers), Materials for 3D printing and additive manufacturing, Nanostructured/nanoscale materials and nanodevices.
- *We identified a bioresorbable material with extraordinary properties. A new material class for active implants was developed, tested and patent submitted*; Stated research areas, Engineering and structural materials, Optical, magnetic and electronic device materials, Bio-inspired, biomedical, and biological materials; nanomedicine, and novel technologies for clinical and medical applications, Materials for energy and environment, next-generation photovoltaics, and green technologies.
- I have prepared a monograph and several scientific articles.
- I conducted literature survey, worked on my pending publications task anabolic attended webinars.
- Not Applicable. However, we need to be patient and careful in difficult time. That is what this pandemic teaches us.
- *Constructed new electrospray instrument*; Stated research areas, Engineering and structural materials, Nanostructured/nanoscale materials and nanodevices.
- None. Only time to finish writing and organizing information.
- I was first able to put my work in order, investigate further, write manuscript on the basis of the acquired results.
- *Assessment on the long-term stability of some of the materials developed for tissue engineering applications and establishment of appropriate conditions for 3D printing of polysaccharide matrices containing cellular spheroids*; Stated research areas, Engineering and structural materials, Organic and soft materials (colloids, liquid crystals, gels, polymers), Materials for 3D printing and additive manufacturing, Bio-inspired, biomedical, and biological materials; nanomedicine, and novel technologies for clinical and medical applications.
- *Me and my colleague work on the materials used for face masks*; Stated research areas, Engineering and structural materials, Nanostructured/nanoscale materials and nanodevices.
- I have improved my software development skill related to the performance prediction of mechanical systems.
- I work on synthesis of polymeric nanofibers that have played a crucial role in manufacture of PPEs.
- Prepared 10 + papers on previous research.
- I applied new mathematical model into thermo-mechanical processing of the pure titanium. It is work based on previous experiments (pre – COVID).
- Super capacitor materials, layered nanostructured materials.
- Hydrogels were prepared by simple condensation of two acids.
- Nothing substantive, but it has been a good time for writing & publishing results
- I discovered that the state of completely online degrees with simulated practical learning is quite advanced.
- I found some mistakes in my lab tests.
- *Sniffer dogs as diagnostics for COVID-19 and extraction of natural products from plants and marine sources for the treatment of cancer*; Stated research areas, Organic and soft materials (colloids, liquid crystals, gels, polymers), Bio-inspired, biomedical, and biological materials; nanomedicine, and novel technologies for clinical and medical applications.
- No, we could not have any new finding since we were writing and discussing the last results, maybe some news in the last experiments already done, but not so much.
- No important lab work was done. However, it was a good opportunity for me to use the time at home to work on previous results and compile and publish them as papers.
- Some guys in group are working, and we got new results about CO and H_2_ evolution using Sn*_3_*O_*4*_/graphene composites.
- The new scientific findings we made during the lockdown period that are related to our research, we are working on the interaction between microbiota and cancer, we found interesting result and we will publish it soon in a new article.
- Less interruptions, more time to write papers and to think.
- *During lockdown, I analyzed data obtained from experimental measurements about a cellulosic compound doped with Ag/AgCl which has photoactivity. This compound can be used for photocatalysis*; Stated research areas, Organic and soft materials (colloids, liquid crystals, gels, polymers), Nanostructured/nanoscale materials and nanodevices, Materials for energy and environment, next-generation photovoltaics, and green technologies.
- It has allowed me and my group to re-visit results and ponder about them, finding in some instances we had overlooked meaningful data which allows to project new or more solid avenues of research.
- *A series of materials were tested in a recognized lab in UK against coronavirus, the results were positives to kill that kind of viruses, for us this was a significant progress in our research agenda*; Stated research areas, Engineering and structural materials, Bio-inspired, biomedical, and biological materials; nanomedicine, and novel technologies for clinical and medical applications.
- Because of the restriction to access to the office, lab facilities, I am mostly focusing on textbook authoring. This solidified my fundamental and theoretical modeling significantly.
- Working on optimization techniques for textile applications. Writing research papers based on left out work during Ph.D work in the area of healthcare textiles.
- Preparation of manuscript for publication.
- *Infrared sensor thermometers developed using 3D technology*; Stated research areas, New topics/Other.
- Automated sample preparation and characterization is highly required.
- 3 PhD finished (supervisor), results published.
- Analysis of data conducted and a number of peer reviewed articles published.
- During lockdown, I wrote 2 Scientifics papers.
- During the lockdown we have published three papers, two book chapters and five papers are going to be submitted.
- Multi-gas identification with hybrid gas sensor system.
- *Medicinal plants effective in reducing the symptoms of COVID-19 with nanotechnology*; Stated research areas, Organic and soft materials (colloids, liquid crystals, gels, polymers), Nanostructured/nanoscale materials and nanodevices, Bio-inspired, biomedical, and biological materials; nanomedicine, and novel technologies for clinical and medical applications.
- Electron phonon coupling at metal/nonmetal interfaces.
- *Development of an improved dyeing process that save water and chemicals without compromising the quality of dyed fabrics. The research was carried out by one of my master’s degree students in a dying factory*; Stated research areas, Organic and soft materials (colloids, liquid crystals, gels, polymers), Nanostructured/nanoscale materials and nanodevices, Bio-inspired, biomedical, and biological materials; nanomedicine, and novel technologies for clinical and medical applications, Materials for energy and environment, next-generation photovoltaics, and green technologies.
- *Prepare a proposal for BRICKS funding on COVID-19*; Stated research areas, Organic and soft materials (colloids, liquid crystals, gels, polymers), Energy materials, Nanostructured/nanoscale materials and nanodevices.
- I had time to carefully analyze the data obtained in the last two years. Statistical data led to important conclusions.
- *Studying about cotton contaminates*; Stated research areas, Organic and soft materials (colloids, liquid crystals, gels, polymers).
- I have prepared several important papers on the base of data processed during lockdown.

Questions: *‘Has your funding been affected by the lockdown?’; What are the major hurdles limiting your research activities?”; ‘Has your current area of research become impossible to continue?’* and *‘Are you considering changing your area of research because of the pandemic?’*

Approximately 21% of respondents said that all their funding was under review and 51% had not received new funding to restart research. Furthermore, insufficient staff (47%) and complicated protocols (54%) were stated as the main difficulties limiting research activities, with 74% saying that their research had not become impossible to continue, although 9% replied ‘yes’ and 14% ‘maybe’ to the same question. This sense of being unable to pursue current research was reflected by the results that 10% ‘maybe’ consider changing research areas, and 8% were definitely going to do so.

Specific comments to the prompt, ‘Has your current area of research become impossible to continue?’

- No access to lab, Reduction in monthly payment, delay in revival of many facilities and no assurance for continuation/renewal of job contracts leads to creation of unhealthy environment which also creates many problems especially monitory deficiency for fulfilling basic need of research scholars and contract basis professor
- It has been difficult for students to work in the laboratory, they need authorization to enter the university. Those students who did not even begin their experimental work are making me worried, will they be able to conclude their master or doctorate? Others can they develop work that does not need laboratory and equipment, only Internet and computers.
- We can spend 30% of our time working at the Institute
- It would be impossible only in case of a prolonged second lock down
- If the pandemic continues by keeping in lock down it would be difficult to make our research freely. So it’s on our minds to take on another field of studies.
- We have access to the lab with appropriate safety measures. Accordingly, my research has continued unabated.
- With social distancing restrictions and personal protective equipment usage we can still conduct practical experiments in the laboratory. This means progress will be significantly slower, but all our researchers continue to work.
- Relaxations in the lockdown have been in place and the activities are slowly getting back on track. Hence, the research activities have resumed.
- I can only go to the lab once a week due to safety protocols, so it is taking so much time.
- Even we have already restarted but very slow, I think is not impossible, but very hard.
- *We are expecting to have access (limited) to the lab in the near future; when and if it happens, we will be able to continue our laboratory-dependent research activities.*
- Research completely stopped without lab access, significantly slowed with restricted access, and more or less resumed with current restrictions. Collaboration with fellow students and other lab groups has all but stopped.
- We increased our R&D efforts during lockdown time, having more time to focus compared to regular industrial routine times.
- I am working on slient coatings, antibacterial coatings also became important in pandemic.
- Specific comments to the prompt, “Are you considering changing your area of research because of the pandemic?”
- My country is under sanctions and I could not change my country simply.
- *One of my areas of expertise is the design of new composite materials with specific properties, years ago we experience with antimicrobial additives which have potential to fight against viruses, so these new findings are so interesting for our lab.*
- I am not thinking to change the seeking research, but only the approaching strategy, from the experimental-oriented to theoretical approach at present.
- I am not sure I can totally change my research area but till the pandemics goes off I may look for more suitable research where i can conduct alone in my home.
- Because of my age, it is impossible to change the field of research.
- Irrespective of lack of sufficient funding, I strongly believe that my research area is very important for human beings.
- I am considering something with less experimentation.
- Yes, I have considered to better do research about new kinds of materials related or applied to prevent COVID-19 in different ways.
- If anything, the pandemic has indicated that we should expand and deepen our areas of interest, currently electrochemical biosensing materials and synthetic procedures to manufacture them.
- *The pandemic has put the importance of my research in perspective, i.e. made it clear that it is not important.*
- We are working on remote area far from crowded people and we can work there.
- Changing to simulations processes instead of lab stuff.

Question: *‘What area(s) of research are likely to emerge and flourish in the post-COVID era?’*

The responses to this question covered a wide range of topics that we have grouped as follows.

1. Health-related research

Keywords: exploring new medicines; pharmacology; virology; vaccine research/development; detection/killing of pathogens; medical biotechnology.

(2) Data science

Keywords: data science; AI; data mining; mobile applications; cloud computing.

(3) Energy-related research

Keywords; storage; lightweight constructions.

(4) Materials-related research

Keywords; additive manufacturing; materials informatics; simulations and computational.

(5) Biology-related research

Keywords: molecular biology; biomaterials; molecular biology.

These results are somewhat surprising because ‘global warming’ and ‘climate change’ were not a high priority for the respondents in this survey.

Question: *‘What have been the most damaging aspects of the Covid-19 pandemic for your research and career?’*

Notable responses were:

- *Delays in upcoming projects and abrupt halt in research.*
- Salary was reduced since working from home.
- No work in the lab possible, no experiments.
- No live conferences, no social interactions with colleagues.
- Budget reduction, future funding unclear, financial problems.
- Unsurprisingly, the overwhelming response to the question, ‘*Have there been any positive outcomes of the COVID-19 pandemic from your perspective?*’ was an unequivocal ‘*No*’. Furthermore, the almost unanimous response to *‘What aspects of your daily research activities have you missed the most since the start of the lockdown?’* was ‘*Meeting people*.’
- Notable general comments about personal experiences related to research during the COVID-19 pandemic:
- Too much administrative workload, including many virtual meetings.
- This may last longer than expected.
- Probably net security will become more serious and hinder research activities.
- Publication procedure has been faster these days.
- We made the best of it. And some outcomes (new project ideas, digital lectures) might not have developed without the restrictions.
- Loss of salary/stipend has become a big problem for many young researchers.
- We have childcare to manage and have been shielding. The stress and exhaustion of that (and at times not being able to get any food) means work has taken less of a priority. The pandemic has meant that we can only do what we really have to do.
- I think we were able to test our adaptability to strong changes in all fields, work, home, food, health care, and we value what we had.
- Due to the travel restriction, I lost foreign visiting researchers from the lab, which reduced our research activities and products.
- People seem to be nicer, help others, care for each other.
- Pandemic = efficiency. The pandemic has shown us that we can cut red tape and unnecessary roads.
- I hope the tools for remote communication become easy-accessible in developing countries. Imagine that if we didn’t have the video conferencing, what would we have done for our communications in this period? I WISH tech providers consider the situation of all students around the world.

**Questions for doctoral students**

*‘What were the immediate effects on your activities when the lockdown was announced?*’

- Shutdown of all my facilities, i.e. no experiments.
- Caring for two kids the whole day, attending online meetings and organizing every day under lockdown conditions shifted the real scientific work into the evening and night time. After some weeks I was not able to keep up this habit, because I could not get enough sleep anymore. The only way out was setting priorities and leaving everything except of priority #1 behind.
- Lack of access to lab -> Difficulties in obtaining an experimental data.
- Virtual working simply increases the time spent in meetings instead of actually doing research.
- Experimental work stopped.
- I continued working and struck back at the shutdown regulations.
- Communication loss
- The work had to stop in midway.
- Planned experiments being blocked.
- Stress and lack of concentration to work, because we did not know what was going to happen.
- Completely missed with my exercise routine which is very hard to start again.
- Fear psychosis after staying indoor for long time.
- I had to interrupt some of my experiments as I suddenly had to stop working without any preparation. So I wasted some of lab materials.
- Long time required to purchase reagents.
- It was very hard time.
- Suspension of all research activities. No access to research data from residence.
- Concern about continuity in research.
- The need to organize new workplace and new teaching system.
- Check all the chemicals that are present in the lab and allotted some days for cleaning purposes.
- All the activities stopped. All projects halted. Prepared samples/products could be stored. Analysis couldn’t be completed.
- Maintenance of lab
- I started to work home.
- All activities ceased for four weeks, while plans to resume safely were being prepared.
- Financial problems
- The labs were completely closed.
- Try to be prepared for virtual teaching.
- Research came to standstill for three months.
- Everything came to zero.
- Interrupted ongoing experiments.
- Go to lab, and shutdown everything in a safe way.
- Managing the backlog work.
- The impossibility to enter the laboratory.
- I have to get back home from my sabbatical leave.
- My study duration was affected due to COVID-19 pandemic situation.
- Restrictions for lab access and reduction in budget for consumables.
- Everything came to an abrupt halt.
- Routine research work and personal life affected.
- Everything stopped.
- Difficulties in submitting new papers.
- University closed and I could not access my notes.
- Isolation.
- Fatigue.
- Difficulty in finding food.
- Became very bored to stay at home and developed stress.
- Everything was shut down.
- Complete halting of all lab work, and shutdown of all instruments for 6 months. Uncertainty of whether they would start up again, in some cases.
- Lack of sleep.
- All research stopped immediately; literature review was the only thing I could do.
- A dark and unknown feeling with fear. It almost paralyzed our day-to-day activities.
- Move to Insilco modelling and time to write up/analyze data backlog
- Decrease of time in the laboratories, but we were not restricted in access. The problem was partially lack of workspace at home for doing daily office activities.
- About 1.5 months of relative inactivity while the world figured out how to do things online
- Fear.

**Figure 8.**
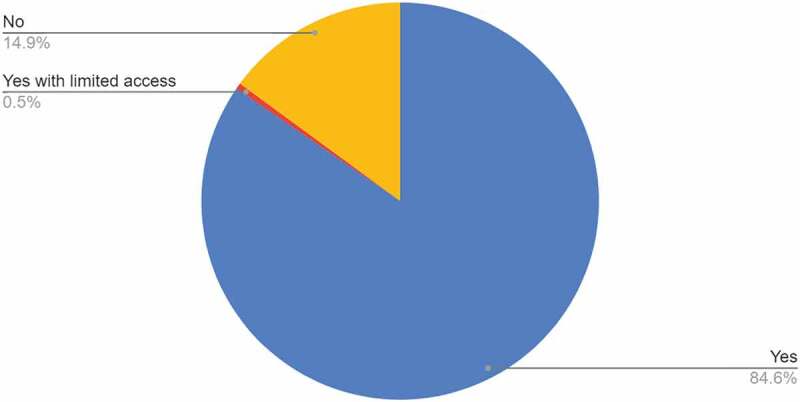
The percentage of doctoral students who were able to restart their research by October 23, 2020

As shown in Figure 8 approximately 85% of the doctoral students were able to restart their research by the deadline for this survey of 23 October 2021 and in response to the question, *‘What are your concerns and worries now?’* the most important issues were related to careers (65%) and restarting research (34%).

## 5. Findings from interviews published in *The STAM Podcasts* [8–10]

The podcasts were produced by Adarsh Sandhu following a generic list of topics and timeline to discuss issues related to research and education during the crisis. The interviewees were sent a list of topics that would be discussed in the conversations. The conversations were spontaneous and the final episodes published in *The STAM Podcast* were only edited for clarity of audio. Importantly, the discussions are snapshots of the thoughts and emotions of the interviewees as they navigated their way through the chaos caused by the spread of coronavirus in their own unique circumstances. The list of topics discussed with all the interviewees is given in Supplementary information 1.

The first conversation was with Professor Atsufumi Hirohata, University of York, UK, and recorded on May 27, 2020. The coronavirus was continuing to spread and the whole of the UK was under hard lockdown, with universities closed to both academics and students. The discussion focussed on the complete stoppage of research activities, merits of online teaching from both sides of the screen, challenges of objective assessment with the new and omnipresent video conference technology, and the consequences for academia if high school students were not able to sit their university entrance exams for 2020. Professor Hirohata’s own local experiences of the difficulties of not being able to do research, virtual conferences, and concerns about doctoral students and their careers concur with the wider global results obtained from our questionnaire.

In contrast to the almost complete lack of mobility and inability to research in the UK, the conversation with Dr Roland Hany at Swiss Federal Laboratories for Materials Science and Technology (Empa) on 9 June 2020, was more relaxed as the spread of coronavirus in Switzerland had been brought under control and Dr Hany was resuming research based on strict safety protocols introduced by his institute. A similar sense of calm was present in discussions with Professor Hong Lin at Tsinghua University in Beijing (June 13, 2020) and Professor Katsuhiko Ariga, NIMS in Japan (June 18, 2020).

But such feelings were not apparent during conversations with Professor Arindam Ghosh, Indian Institute of Science, Bangalore, India (June 22, 2020) and Professor Daniel Ortega Ponce, Universidad de Cádiz/IMDEA Nanoscience, Spain (June 24, 2020), where their institutes were still under strict lockdown.

The conversation with Professor James K. Gimzewski, University of California, Los Angeles (July 2020), offered his deep insights into the social issues engulfing the USA at the time. And notably, the discussion also highlighted how materials scientists were continuing to adapt and innovate to overcome the severe limitations to personal mobility. Specifically, Professor Gimzewski shared his views about establishing global robot-based hubs to perform experiments on chemical synthesis for both research and education by coupling state-of-the-art machine control technology with telecommunications.

## 6. Conclusions

Now, as we write this paper in mid-Jan 2021 the Covid-19 coronavirus continuous to spread across the world. The development, approval, and roll out of vaccines offers hope as another wave of lockdowns are implemented worldwide amidst concerns about the discovery of variants of Covid-19 in the UK and South Africa.

Our survey was carried out during the first wave of global lockdowns covering the period March to October 2020, when research institutes initially shut down and some were able to restart activities in late June. Some countries in Asia were able to quickly control the spread of coronavirus thereby enabling researchers and grad-students to restart their research between June and August. *The STAM Podcast* interviews recorded between June and September 2020 with researchers in China and Japan offer direct insights into the situation in these countries at the time, and the conversations are in sharp contrast to those with materials scientists based in the US, India, and Europe, where there were still severe lockdowns [8–11].

The survey showed the materials science community to be resilient and adaptive to overcome limitations imposed by restrictions to mobility as exemplified by the proposal for ‘robot-based hubs’ to perform remote chemical synthesis similar to astronomers who use remote control to move massive telescopes located all over the world.

Responses from doctoral students highlighted their ‘fear’ and uncertainty as their work suddenly came to a halt and they worried about their careers. But grad-students also learnt to move forward by using their time to reassess previous data with a view to publishing papers and planning their research.

We hope that the results of this survey will offer a timely resource for the materials science community to learn from each other’s experiences and be able to adapt and move forward from chaos inflicted on their research by the spread of the Covid-19 coronavirus.

Unfortunately, almost one year after the first confirmed cases of Covid-19 outside of China, many of us are once again under strict lockdown, but with the glimmer of hope that mass vaccinations will bring relief to our plight. As the world moves into 2021, we want to continue collate and share the experiences of scientists in the materials science community and are planning to conduct another survey later this year to update our findings for 2020.

## Supplementary Material

Supplemental Material

Supplemental Material
