# Supplementary material for: Global snapshot of the effects of the COVID-19 pandemic on the research activities of materials scientists between Spring and Autumn 2020
Source: Sci Technol Adv Mater. 2021 Apr 21;22(1):173–84. doi: 10.1080/14686996.2021.1894756 (PMC8079126; doi:10.1080/14686996.2021.1894756)
Supplement: Supplemental Material [file TSTA_A_1894756_SM5428.docx]

**Supplementary information 2**

**List of topics discussed with all the interviewees for the *STAM Podcast***

I. Outline of research activities. Motivation, goals, recent findings and their social implications.

II. What were your thoughts between January to March 2020 after the initial reports of the Covid-19 coronavirus outbreak in Wuhan but prior to the implementation of lockdown measures? Were you and your institute prepared for possible such crises lockdowns?

III. What happened in your particular institute when lockdown measures were initiated? Establishing a crisis center/ Policy committees/Efficiency of disseminating information on guidelines to staff/students/ Effective use of information technology/lectures/research equipment/grad students/post-docs and other staff.

IV. How did your thoughts change as the lockdown was extended? Daily routines/Interaction with colleagues and other members of your group.

V. Teaching: If you teach, then how are you finding on-line teaching. Learning to use web video conferencing technology/ Did your institute provide instructions/How are you managing experimental labs and tutorials where normally face to face is essential? Are the students satisfied with on-line lectures/Value for money/ Assessment.

VI. If you are focused on research only, without teaching obligations, how has your world changed? Concerns over leaving equipment idling, incomplete experiments, staying motivated.

VII. How much ‘damage’ has the pandemic done to your research activities? Funding/waste materials resources perishable items/ Recruiting grad-students/post docs.

VIII. Specific plans when restrictions are relaxed? Worries and concerns/ Or perhaps, an opportunity to try something completely different.

IX. Positive aspects of the lockdown? Able to concentrate on writing papers/ developing new ideas/learn the piano. History records that Isaac Newton retreated to his Lincolnshire home during the pandemic in 1665, which yielded some of his seminal work.

X. How have you managed your health during the crisis?

XI. Anything else.
