# Supplementary material for: Global snapshot of the effects of the COVID-19 pandemic on the research activities of materials scientists between Spring and Autumn 2020
Source: Sci Technol Adv Mater. 2021 Apr 21;22(1):173–84. doi: 10.1080/14686996.2021.1894756 (PMC8079126; doi:10.1080/14686996.2021.1894756)
Supplement: Supplemental Material [file TSTA_A_1894756_SM5387.docx]

***
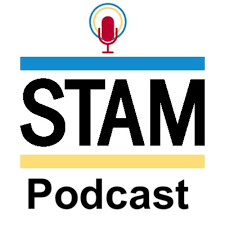
Supplementary information 1. List of interviewees for The STAM Podcast***

Apple Podcast

<https://href.li/?https://podcasts.apple.com/podcast/the-stam-podcast/id1517265384>

The latest news, views, and research highlights from the editors of the journal "Science and Technology of Advanced Materials" for the global community of materials scientists.

Jan 18, 2021

Jung Ho Kim: Materials research, education, and covid-19 (Australia/Recorded 9 December 2020)

In conversation with Professor Jung Ho Kim, Australian Institute for Innovative Materials University of Wollongong, Australia, about his observations of materials research and society in Australia during the restrictions triggered COVID-19.

31 min

Jan 17, 2021

Pooi See Lee: Materials research, education, and covid-19 (Singapore, Recorded 4 December 2020)

In conversation with Professor Pooi See Lee, Nanyang Technological University, Singapore about her observations of materials research and society in Singapore, during the restrictions triggered by COVID-19.

32 min

Dec 28, 2020

Fabien Grasset: Materials research, education, and covid-19 (France/Recorded 23 November 2020)

In conversation with Professor Fabien Grasset, Director of research at ISCR, Rennes, France, about his observations of materials research and society in France during the restrictions triggered by COVID-19.

37 min

Sep 14, 2020

James K. Gimzewski: Materials research, education, and covid-19 (USA/4 July 2020)

In conversation with Professor James K. Gimzewski, University of California, Los Angeles, about his observations of materials research and society in the US during the restrictions triggered by COVID-19.

24 min

Aug 17, 2020

Daniel Ortega Ponce: Materials research, education, and covid-19 (Spain/24 June 2020)

In conversation with Professor Daniel Ortega Ponce, Universidad de Cádiz/IMDEA Nanoscience, Spain, about his observations of materials research and society in Spain during the restrictions triggered by COVID-19.

Jul 21, 2020

Arindam Ghosh: Materials research, education, and covid-19 (India/Recorded 22 June 2020))

In conversation with Professor Arindam Ghosh, Indian Institute of Science, Bangalore, India, about his observations of research and society in India during the restrictions triggered by COVID-19.

23 min

Jul 7, 2020

Katsuhiko Ariga: Materials research, education, and covid-19 (Japan/Recorded 18 June 2020))

In conversation with Professor Katsuhiko Ariga, NIMS in Japan, about his observations of research and society in Japan during the restrictions triggered by COVID-19.

17 min

Jul 4, 2020

Hong Lin: Materials research, education, and covid-19 (China/Recorded 13 June 2020)

In conversation with Professor Hong Lin at Tsinghua University in Beijing, about her observations of research and society in China during the restrictions triggered by COVID-19.

26 min

Jul 1, 2020

Roland Hany: Materials research, education, and covid-19 (Switzerland/Recorded 9 June 2020))

In conversation with Dr Roland Hany at Empa in Switzerland, about his observations of the effects of the COVID-19 pandemic on research and society in Switzerland.

17 min

Jun 7, 2020

Atsufumi Hirohata: Materials research, education, and covid-19 (UK/Recorded 27 May 2020))

In conversation with Professor Atsufumi Hirohata, University of York, UK, about the unique challenges facing materials scientists as they assess the impact of the unprecedented changes triggered by COVID-19.

27 min
